# Disproportionate Contribution of Right Middle Lobe to Emphysema and Gas Trapping on Computed Tomography

**DOI:** 10.1371/journal.pone.0102807

**Published:** 2014-07-23

**Authors:** Surya P. Bhatt, Jessica C. Sieren, John D. Newell, Alejandro P. Comellas, Eric A. Hoffman

**Affiliations:** 1 Division of Pulmonary and Critical Care Medicine, University of Iowa, Iowa City, Iowa, United States of America; 2 Departments of Radiology and Biomedical Engineering, University of Iowa, Iowa City, Iowa, United States of America; Clinica Universidad de Navarra, Spain

## Abstract

**Rationale:**

Given that the diagnosis of chronic obstructive pulmonary disease (COPD) relies on demonstrating airflow limitation by spirometry, which is known to be poorly sensitive to early disease, and to regional differences in emphysema, we sought to evaluate individual lobar contributions to global spirometric measures.

**Methods:**

Subjects with COPD were compared with smokers without airflow obstruction, and non-smokers. Emphysema (% low attenuation area, LAA_insp_<−950 HU, at end-inspiration) and gas trapping (%LAA_exp_<−856 HU at end-expiration) on CT were quantified using density mask analyses for the whole lung and for individual lobes, and distribution across lobes and strength of correlation with spirometry were compared.

**Results:**

The right middle lobe had the highest %LAA_insp_<−950 HU in smokers and controls, and the highest %LAA_exp_<−856 HU in all three groups. While RML contributed to emphysema and gas trapping disproportionately to its relatively small size, it also showed the least correlation with spirometry. There was no change in correlation of whole lung CT metrics with spirometry when the middle lobe was excluded from analyses. Similarly, RML had the highest %LAA_exp_<−856 HU while having the least correlation with spirometry.

**Conclusions:**

Because of the right middle lobe’s disproportionate contribution to CT-based emphysema measurements, and low contribution to spirometry, longitudinal studies of emphysema progression may benefit from independent analysis of the middle lobe in whole lung quantitative CT assessments. Our findings may also have implications for heterogeneity assessments and target lobe selection for lung volume reduction.

**Clinical Trial Registration:**

ClinicalTrials.gov NCT00608764

## Introduction

Airflow limitation in chronic obstructive pulmonary disease (COPD) is due to partially reversible narrowing and dynamic expiratory collapse of airways secondary to emphysema. Currently, the diagnosis of COPD rests on demonstrating airflow limitation by spirometry.[1] However, spirometry is poorly sensitive to early changes, and to regional differences in the distribution of emphysema and air trapping. By means of automated densitometric analyses, computed tomography (CT) can quantify the extent of emphysema, correlates well with pathology,[2] and has been proposed as a mode to diagnose emphysema in its early stages.[3], [4] Multiple studies have shown a correlation between spirometry and CT metrics of emphysema,[5], [6], [7], [8], [9], [10], [11], [12] and CT densitometry might be more sensitive in detecting emphysema than spirometry, likely due to a marked heterogeneity of the distribution of emphysema.[6], [13]

Emphysema in regions that contribute a lesser amount might be less detectable by spirometry. Airflow limitation measured by the forced expiratory volume in the first second (FEV_1_), and the ratio of FEV_1_ to the forced vital capacity (FVC) (FEV_1_/FVC), correlate best with lower zone predominant emphysema.[14], [15], [16], [17] There is also a differential effect of involvement of central compared to peripheral zones on lung function tests.[14] Most of these studies relied on arbitrarily defined zones and areas of delineation, and were also limited by analyses of predefined cross-sectional images that might not have been truly representative of the entire lung. An accurate description of the relative distribution of emphysema is important for diagnosis and physiologic characterization. Recent advances in CT quantification of emphysema and lung segmentation have made it possible for us to study emphysema distribution by lobe, and determine the relative contribution to functional impairment measured by spirometry. Given the heterogeneity, we hypothesized that some lobes of the lung might be “hidden” and remain silent on spirometry in early emphysema. To answer this question, we compared subjects with COPD, smokers without COPD and a normal cohort of young never-smokers.

## Materials and Methods

This research protocol was approved by the institutional review board at the University of Iowa (IRB#200710717 and 199708651), and all participants provided written informed consent. More details of patient selection and imaging protocols are provided in File S1.

### Subjects

#### Cases

Participants in the COPDGene study enrolled from a single imaging center at the University of Iowa were included. COPDGene is a multicenter study that enrolled subjects between the ages of 45 and 80 years with at least 10 pack-years of cigarette smoking. Details of the study protocol have been published previously.[18] Briefly, we included subjects who met the GOLD (Global Initiative for Chronic Obstructive Lung Disease) criteria for COPD, and subjects at risk for COPD, smokers without airflow obstruction on spirometry (FEV_1_/FVC>0.70; FEV_1_≥80% predicted)[1].

#### Controls

Normal non-smokers from a second study that prospectively obtained CT data (Image and Model Based Analysis of Lung Disease: NIH HL-064368) at the University of Iowa were included. Subjects were normal healthy volunteers between the ages of 20 and 90 years, who smoked not more than 20 cigarettes in their lifetime, and had normal spirometry.

### Imaging

CT data from both normal subjects as well as COPDGene subjects were collected were obtained from a single Siemens Somatom Sensation 64 CT scanner (Siemens Healthcare, Erlangen, Germany) residing within a dedicated pulmonary imaging research facility. For COPDGene subjects, the imaging protocol consisted of slice thickness of 0.75 mm, slice spacing of 0.5 mm, and pitch of 1.1. Exposure parameters were 120 kV and rotation of 0.5 seconds, and 200 mAs for inspiratory scans and 50 mAs for expiratory scans. For controls, slice thickness ranged from 0.75 to 1.3 mm, slice spacing was 0.5 to 0.6 mm, and the pitch range was 1.0 to 1.5. Exposure parameters were 100 mAs, 120 kV, and rotation of 0.5 seconds. A Bf31 kernel was used for all groups. Images were analyzed using the Apollo software (VIDA Diagnostics, Coralville, IA).[19] Lobar segmentation was achieved using an anatomy guided graph search method incorporated into the analysis software (Pulmonary Workstation, VIDA Diagnostics).[20] As severe emphysema can challenge automated lobar segmentation, a rigorous quality control process combined with manual correction when required was employed to finalize lobar segmentation. The total segmented lung volumes, as well as the lobar boundaries, at end inspiration (at total lung capacity, TLC) and at end expiration (spirometrically determined 20% vital capacity in the case of normal subjects and residual volume, RV, in the case of COPDGene subjects) were assessed.[21] Percentage emphysema was calculated using the percentage of lung or lobe volume at TLC with attenuation less than −950 Hounsfield Units (HU). These were low attenuation areas (LAA<−950 HU, LAA950_insp_). The percentage of gas trapping was calculated using the percentage of lung or lobe volumes on expiratory scans with attenuation less than −856 HU (LAA856_exp_). Lobar predominance was defined by the lobe with the maximum %LAA950_insp_ for emphysema and %LAA856_exp_ for gas trapping.

### Spirometry

Spirometry was performed according to the American Thoracic Society (ATS) guidelines.[22] Post bronchodilator values were used for diagnosis of COPD using a fixed cut-off of FEV_1_/FVC of <0.70.

### Statistical Analyses

Baseline characteristics were compared by one-way Analysis of Variance (ANOVA) using Tukey’s test for post hoc comparisons between groups. Analysis of Co-Variance (ANCOVA) was used to adjust for the effect of age, gender, height and BMI on CT measures of emphysema, with Bonferroni correction for multiple comparisons. Correlation between %LAA950_insp_ and %LAA856_exp_ with spirometric variables was assessed using Pearson’s correlation test. Correlation was performed for %LAA for the whole lung, as well as for individual lobes. Differences in correlation between spirometry and lobar measures of %LAA across lobes were calculated with Steiger’s Z test for correlated correlations using FZT computator. In each subject, absolute %LAA950_insp_ was compared between individual lobes using repeated measures ANOVA. The lobe with the maximum %LAA950_insp_ was deemed the dominant lobe. Similar analyses were repeated for %LAA856_exp_ gas trapping. The right middle lobe (RML) dominance was assessed across GOLD COPD stages as well as in smokers and controls. p value<0.05 was deemed statistically significant. All analyses were performed using Statistical Package for the Social Sciences (SPSS 11.5, SPSS Inc., Chicago, IL, USA).

## Results

We included 477 subjects (Table 1). Subjects with COPD and smokers without COPD had similar ages whereas controls were significantly younger. COPD subjects had a higher smoking burden, with a significant correlation between number of pack-years and %LAA950_insp_ (r = 0.19; p<0.01) and %LAA856_exp_ (r = 0.29; p<0.01). %LAA950_insp_ and %LAA856_exp_ also increased with age (r = 0.16 and 0.54 respectively; p<0.01). Of those with COPD, 48 had GOLD (Global Initiative for Chronic Obstructive Lung Disease) [23] grade I (27.3%), 87 had grade II (49.4%), 30 had grade III (17.0%), and 11 (6.3%) had grade IV airflow obstruction.

**Table 1:** Comparison of baseline characteristics.

Variable	COPD (n = 176)	Smokers (n = 217)	Non smokers (n = 84)
Age (years)	66.9 (6.9)**: Range45 to 80 years	62.6 (8.1)**; Range45 to 78 years	34.6 (15.1); Range20 to 80 years
Sex, female (%)	77 (44)	109 (50)	34 (40)
BMI (kg/m^2^)	29.0 (5.8)**	30.0 (6.1)**	25.0 (3.8)
Pack-years	58.2 (26.7)**	41.3 (21.7)**	-
Current smokers (%)	55 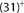	60 (28)	-
FEV_1_/FVC	55 (12)**	78 (5)**	82.2 (5.5)
FVC%	90.7 (17.9)**	95.2 (10.7)**	104.5 (11.5)
FEV_1_%	67.2 (21.0)**	97.8 (11.0)**	109.6 (13.1)
MMRC	1.3 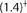	0.4 (0.7)	-
TLC by CT (liters)	6.4 (1.4)*	5.9 (1.1)	5.9 (1.1)
FRC by CT (liters)	3.6 (1.0)**	2.7 (0.6)	2.9 (0.7)

All values expressed as Mean (standard error, SE). BMI = Body mass index; FEV_1_ = Forced expiratory volume in 1 second; FVC = Forced vital capacity; MMRC = Modified Medical Research Council Dyspnea Scale; BODE = Body-Mass Index, Airflow Obstruction, Dyspnea, and Exercise Capacity Index; TLC = Total lung capacity; FRC = Functional residual capacity.

**significant difference compared to non smokers at p<0.001.

*significant difference compared to non smokers at p<0.01.

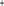
significant difference compared to smokers at p<0.001.

### Quantification of CT densitometry (%LAA950_insp_ and %LAA856_exp_)

The mean (standard error, SE) %LAA950_insp_ for the entire lung was 13.3(10.5)% in the population with COPD. As expected, this was higher than in smokers without COPD and controls (Table 2). This relation held true when assessed for individual lobes of the lung (Table 2). On comparing the %LAA950_insp_ between lobes, the highest percentage was seen in the right middle lobe (RML) in controls and smokers (p<0.01). This was not observed in COPD as there was higher %LAA950_insp_ in the upper and lower lobes (Table 2). However, on comparing %LAA856_exp_, a measure of small airways disease and gas trapping, there was a significantly higher degree of LAA856_exp_ in RML across all three groups. When lobes were graded by the highest degree of %LAA950_insp_, RML had the highest percentage in 42% of subjects with COPD. In comparison, RML had the highest %LAA950_insp_ in 66% of smokers and 75% of controls. The RML %LAA950_insp_ predominance declined in smokers, and with increasing COPD stage (44%, 45% and 32% respectively for stages 1, 2 and combined 3&4). When lobes were graded by the highest degree of %LAA856_exp_, RML had the highest percentage in 72% of subjects with COPD, and in 99% of both smokers and controls. The RML %LAA856_exp_ predominance declined with increasing COPD stage (83%, 76%, and 49% respectively for stages 1, 2 and combined 3&4).

**Table 2:** Comparison of CT measures of emphysema and air trapping.

Variable		COPD(n = 176)	Smokers(n = 217)	Non smokers(n = 84)	Correlation with FEV_1_/FVC(COPD, Smokers, Non-smokers)
Total	LAA950_insp_	13.3 (10.5)* ¥	5.7 (3.3)*	7.9 (4.2)	−0.76; −0.32; −0.31
	LAA856_exp_	33.4 (17.2)* ¥	13.7 (7.8) ¥	12.9 (10.6)	−0.83; −0.36; −0.39
Left Lower Lobe	LAA950_insp_	12.4 (10.6)* ¥	5.6 (3.1)	7.5 (4.2)	−0.71; −0.29; −0.27
	LAA856_exp_	25.8 (18.4)* ¥	7.0 (5.2)	5.2 (6.4)	−0.81; −0.39; −0.32
Left Upper Lobe	LAA950_insp_	14.3 (10.9)* †	6.3 (4.1)* †	9.3 (4.8)	−0.72; −0.31; −0.30
	LAA856_exp_	37.7 (17.8)*	16.8 (10.5)¥	17.8 (14.4)	−0.75; −0.33; −0.38
Right Lower lobe	LAA950_insp_	11.9 (10.4)* †	5.3 (3.2)	6.9 (4.0)	−0.72; −0.30; −0.28
	LAA856_exp_	25.7 (18.4)* ¥	7.2 (5.1)	4.5 (6.9)	−0.82; −0.37; −0.26
Right Middle Lobe	LAA950_insp_	14.3 (10.7)*	8.1 (4.9)* †	11.1 (5.5)	−0.63; −0.26; −0.31
	LAA856_exp_	46.5 (17.5)*	28.9 (13.2)¥	32.8 (17.7)	−0.67; −0.29; −0.34
Right Upper Lobe	LAA950_insp_	13.6 (13.3)* ¥	4.5 (4.0)	6.5 (3.8)	−0.70; −0.28; −0.32
	LAA856_exp_	36.2 (19.4)* †	15.3 (10.2)¥	12.6 (13.5)	−0.79; −0.33; −0.37

All values expressed as Mean (standard deviation, SD). LAA950insp = Percent Low attenuation area below −950 HU at end inspiration. LAA856exp = Percent Low attenuation area below −856 HU at end expiration.

*significant difference on univariate analysis compared to non smokers at p<0.01.

† significant difference compared to non smokers on multivariate analysis, adjusted for age, gender, height and body mass index, at p<0.05.

¥ significant difference compared to non smokers on multivariate analysis, adjusted for age, gender, height and body mass index, at p<0.001.

### Correlation of CT densitometry with spirometry

**Figure 1:**
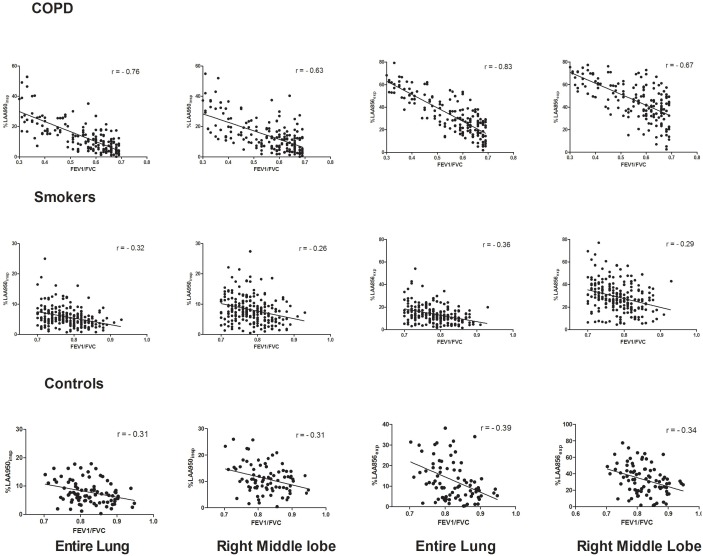
Correlation (Pearson’s correlation r) between the FEV_1_/FVC ratio with emphysema-like changes (%LAA<−950 HU at end inspiration) and gas trapping (%LAA<−856 HU at end expiration), for the whole lung and separately for the right middle lobe. FEV_1_ = Forced Expiratory Volume in the first second. FVC = Forced Vital Capacity. LAA = Low Attenuation Areas. HU = Hounsfield Units. p<0.05 for all correlations. Right middle lobe showed significantly less correlation for FEV_1_/FVC (p<0.01).

**Figure 2:**
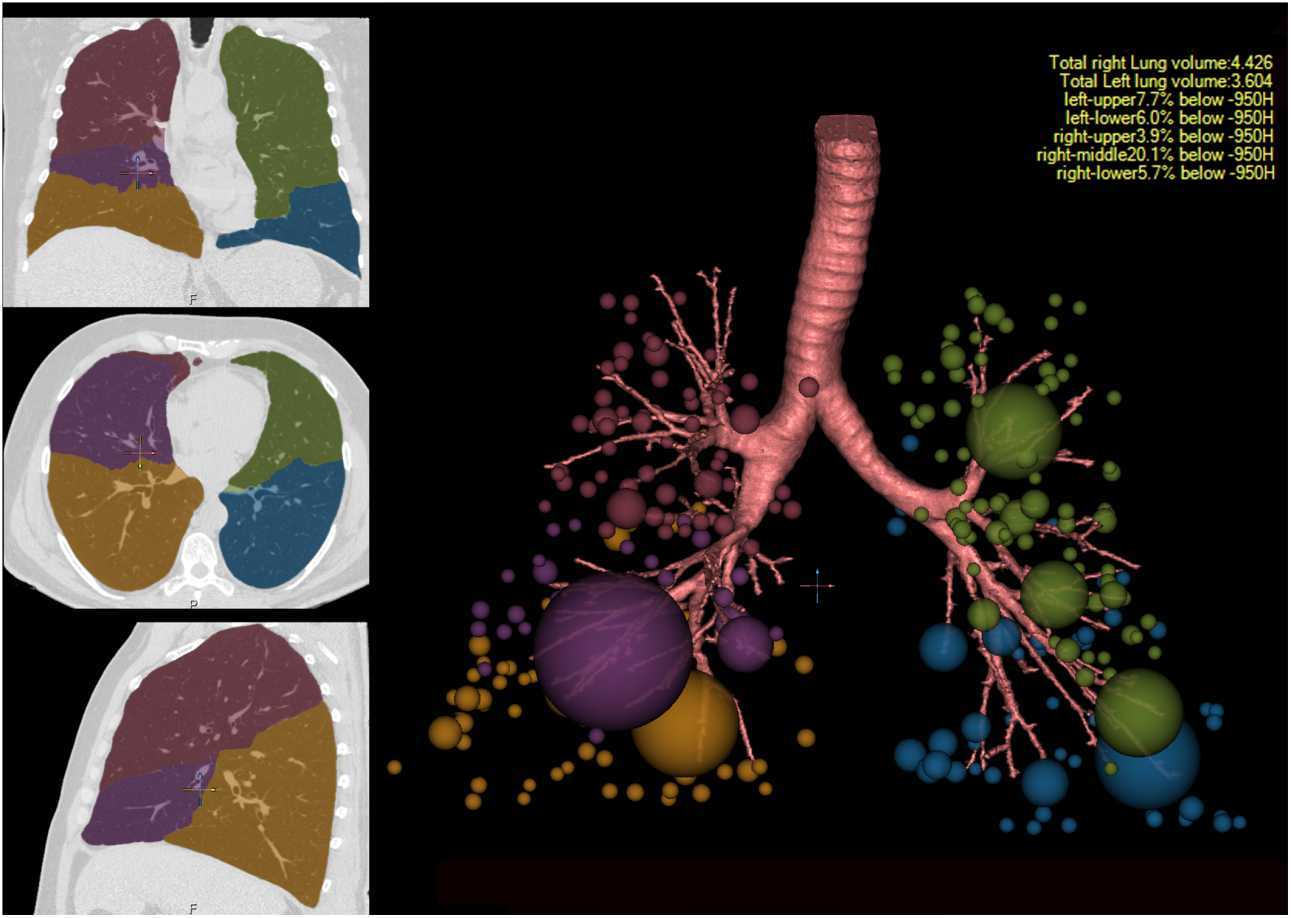
Example of a CT scan obtained from a GOLD 0 smoker with FEV_1_ %predicted of 106 and an FEV_1_/FVC ratio of 0.78. As seen in the left panel, the lungs were segmented, via VIDA’s Apollo Software, automatically with automated delineation of the individual lung lobes (Right Upper Lobe: Pink; Right Middle Lobe: Purple; Right Lower Lobe: Yellow; Left Upper Lobe Green, Left Lower Lobe: Blue). In the right panel, the distribution of the clustered regions of emphysema-like lung are shown as spheres demonstrating the cluster size expressed as sphere diameter and the centroid of the clustered region. Emphysema-like lung was defined at voxels below −950 HU. Spheres are color coded similarly to the coding in the left panel to distinguish lobar locations. The lobar distribution of percent emphysema-like lung is tabulated in the upper right portion of the right panel. Note that the right middle lobe shows significantly greater presence of emphysema-like lung as compared with the other lobes. However, the PFTs are more reflective of the percent emphysema in all lobes other than the right middle lobe.

The %LAA950_insp_ in COPD correlated well with airflow obstruction (r for FEV_1_/FVC = −0.76; Figure 1). There was also a significant correlation between spirometry and %LAA950_insp_ in individual lobes (r = −0.63 to −0.75; p<0.01) (Table 2). However, RML correlated less with spirometry than did other lobes (r = −0.63; Steiger’s Z = 4.03; p for difference in correlation <0.01). Figure 2 shows a representative example of a subject with disproportionate emphysema-like changes that did not get reflected in spirometry. While the smaller volume of RML could have accounted for this, it correlated less even after adjustment for the size of individual lobes by CT volume (r = −0.52 vs. −0.62 to −0.71 for other lobes; p for difference in correlation <0.01).

The %LAA856_exp_ in COPD correlated well with airflow obstruction (r for FEV_1_/FVC = −0.83; Figure 1). There was also a significant correlation between spirometry and %LAA856_exp_ in individual lobes (r = −0.67 to −0.82; p<0.01). However, RML correlated less with spirometry than did other lobes (r = −0.67; Steiger’s Z = 7.32; p for difference in correlation <0.01) (Figure 1).

The impact of this observation would be unclear without an assessment of relative contribution of RML to the total volume of the lung as assessed by CT volumetry, and its contribution to total %LAA950_insp_ and %LAA856_exp_. TLC was 5.9 to 6.4 liters across the three groups (Table 1), of which 8% was comprised of the RML. Conversely, RML contributed to 12%, 12% and 9% respectively of %LAA950_insp_ in controls, smokers and subjects with COPD. Similar analysis of gas trapping was more revealing. FRC was 2.7 to 3.6 liters (Table 1), of which RML contributed 10% in controls and smokers, and 9% in COPD. In contrast, the relative contribution of RML to total gas trapping was 34%, 24% and 15% respectively (p<0.01). Table 3 shows a comparison of correlation of CT metrics with FEV_1_/FVC for the whole right lung, and after subtracting RML. There was no difference in correlation, again demonstrating the small effect of RML on spirometry, despite the higher %LAA950_insp_ changes and %LAA856_exp_ seen in this lobe, adjusted for lobar volume (p for difference in correlation <0.01; Table 2).

**Table 3:** Correlations between CT measures and FEV_1_/FVC for Right Lung with and without middle lobe.

		Right Lung (Whole)	Right Lung minus RML	RML
LAA950insp	COPD	−0.73	−0.73	−0.49
	Smokers	−0.31	−0.31	−0.24
	Controls	−0.34	−0.31	−0.38
LAA856exp	COPD	−0.82	−0.83	−0.51
	Smokers	−0.34	−0.34	−0.29
	Controls	−0.35	−0.31	−0.38

All are Pearson’s r values. LAA950insp = Percent Low attenuation area below −950 HU at end inspiration. RML = Right Middle Lobe. LAA856exp = Percent Low attenuation area below −856 HU at end expiration.

## Discussion

Two key observations are presented. First, RML shows elevated %LAA950_insp_ and %LAA856_exp_ even in young non-smokers. Second, even though with increasing severity of COPD, the upper and lower lobes show proportionally more %LAA950_insp_ and %LAA856_exp_, RML continues to present with the greatest values for each measure, and contributes to total lung density disproportionate to its relatively small size. Despite this, there is poorer correlation of RML with spirometric measures compared to other lobes. This suggests that RML changes can remain relatively hidden from detection by spirometry.

The GOLD COPD guidelines recommend spirometry for diagnosis and staging of severity.[1] However, Omori et al. showed that a third of current smokers showed evidence of emphysema on CT, but 3/4ths of these subjects had normal spirometry.[24] Because of the heterogeneity of disease in COPD, automated CT measures of emphysema and gas trapping are increasingly being used to identify discrete phenotypes. Most quantitative CT volumetric studies of emphysema have been done on subjects with COPD,[6], [12], [25] or smokers without airway obstruction,[26] or normal controls alone,[27] and to our knowledge, this study is the largest comparison of emphysema quantification between COPD and controls.

With CT being performed increasingly to supplement spirometry in the early diagnosis of emphysema, it is important to define the relative distribution of LAA in subjects with COPD and normal controls. We showed that RML has the greatest percentage of %LAA950_insp_ changes (%LAA950_insp_) and gas trapping (%LAA856_exp_) in normal controls and smokers. With increasing severity of COPD, the upper and lower lobes gradually surpass the RML, resulting in a lesser contribution from RML. However, RML retains predominance in a majority of COPD subjects. The cross sectional nature of data collection prevents us from concluding that this happens in every subject with COPD.

The basis for RML presenting with a greater degree of %LAA950_insp_ changes and %LAA856_exp_ is unclear. This lobe is unique in its susceptibility to injury, often being involved when other lobes are spared.[28] On the other hand, since these RML-derived measures are elevated in the normal subject, caution should be exercised when concluding that elevated measures indicate a greater susceptibility of the RML to pathology. Taking into consideration the RML differences in normal subjects and susceptibility of the RML to injury, preferential disease progression could be related to a differential deposition of inhaled particles.[29], [30] While studies have shown that particle deposition is least in RML compared to other lobes, it is also possible that egress of particles once deposited is also lowest in the RML due to anatomic disadvantages.[29], [30] We speculate that this might be due to the distinct anatomic features of RML. Computational fluid dynamics approaches to the evaluation of CT-derived lung data may offer new insights.[31] The lobar bronchus is relatively narrow with an acute take-off from the bronchus intermedius,[32] possibly predisposing this lobe to more gas trapping with equivalent degrees of emphysema. Gas trapping, in turn, can affect estimation of emphysema by contributing to a lower density over subsequent respiratory cycles.[33] RML also has poor collateral ventilation when the fissures are complete due to the large pleural-surface-area to lobar-volume ratio.[34] Collateral ventilation is important in homogenization of ventilation; and, while lack of collaterals is commonly thought to be important in the mechanism of atelectasis and middle lobe syndrome, this might also be important in over-inflation. Collaterals in the RML have a higher resistance to airflow and the time constant for collateral ventilation in RML far exceeds that in other lobes,[34], [35] and over time, this might result in a greater degree of gas trapping. The RML predominance of %LAA950_insp_ is, to our knowledge, a new finding, and the above remains conjectural.

Airway branches were excluded before assessment of LAA, but the relative density of smaller branches beyond the 5th generation might be higher in RML, affecting LAA measurements. We also considered the effects of gravity as there can be a vertical gradient in lung density with dependent areas showing a higher density.[36] However, this should have affected the upper lobes as well, since they are preferentially non-dependent relative to the lower lobes.

There are a number of clinical implications of these findings. That RML densitometry correlates least with airflow obstruction, despite significant contribution to overall gas trapping which in turn correlates strongly with spirometry, suggests that it can remain “hidden’ from detection by spirometry. Furthermore, disproportionate contribution of RML to overall lung destruction scores and progressively lower contribution with increasing emphysema suggests longitudinal studies will be affected by an underestimation of rate of disease progression due to relatively low contribution from this lobe. Our findings suggest that caution should be exercised when interpreting LAA percentages, and perhaps the RML should be analyzed independent of the other lobes. Lastly, RML is frequently used for assessment of heterogeneity prior to bronchoscopic lung volume reduction procedures, but is excluded as a target lobe for intervention due to its relatively small size. Our findings of disproportionate and significant contribution to gas trapping may have implications for selection of patients for biologic lung volume reduction.

Our study has a number of strengths. The CT scans were done at a single center using a standardized protocol on the same scanner. This eliminated scanner variation that can be as large as 10 to 20%.[37] The automated determination of emphysema eliminated inter- and intra-observer variability associated with visual scoring of emphysema. Lastly, the assessment of densitometry by lobe provides more anatomically useful information. Our results seem to show that even though smokers had more gas trapping than normal controls, they appeared to have lower %LAA950_insp_. Recent studies have shown that smoking increases lung density,[38] and this could serve to erroneously reduce %LAA950_insp_. Our study also has some limitations. Almost all subjects (99% of COPD and smokers; 88% of controls) were Caucasian. There are significant racial differences in spirometry and it is unclear how this will affect correlations with emphysema distribution. Controls were significantly younger than cases, but all comparisons were adjusted for age. Finally, subjects were enrolled from a single center. However, the distribution of subjects by GOLD staging appears to be representative of the usual COPD population.

In conclusion, our findings demonstrate that the increased RML presence of baseline %LAA950_insp_ and %LAA856_exp_ that has been observed in normal non-smokers persists in COPD subjects. However, the persistent increased %LAA950_insp_ (emphysema-like) lung and %LAA856_exp_ (“gas trapping”) of the RML is silent to overall pulmonary function tests. Because of RML’s disproportionate contribution to CT based density measurements, and low contribution to spirometry, longitudinal studies of emphysema progression may benefit from the separate evaluation of RML relative to whole lung quantitative CT assessments. Our findings may also have implications for heterogeneity assessments and target lobe assessments for bronchoscopic lung volume reduction procedures.

## Supporting Information

File S1 (DOC)
