# Supplementary material for: Disproportionate Contribution of Right Middle Lobe to Emphysema and Gas Trapping on Computed Tomography
Source: PLoS One. 2014 Jul 23;9(7):e102807. doi: 10.1371/journal.pone.0102807 (PMC4108372; doi:10.1371/journal.pone.0102807)
Supplement: File S1 — (DOC) [file pone.0102807.s001.doc]

**Supplement:**

**Methods:**

This research protocol was approved by the institutional review board at the University of Iowa (IRB# 200710717 for COPDGene, and 199708651 for the Biomedical Research Partnership (BRP) study, and all participants provided written informed consent.

**Subjects: *Cases:*** Participants in the COPDGene study enrolled from a single imaging center at the University of Iowa were included. COPDGene is a multicenter study which enrolled subjects between the ages of 45 and 80 years with at least 10 pack-years of cigarette smoking. Details of the study protocol have been published previously. Briefly, we included subjects who met the GOLD (Global Initiative for Chronic Obstructive Lung Disease) criteria for COPD. We also studied a second group of subjects who were deemed to be at risk for COPD. These were smokers without airflow obstruction on spirometry (FEV_1_/FVC >0.70; FEV_1_ >80% predicted). Subjects were either of non-Hispanic Caucasian or African-American descent. Major exclusion criteria included other lung diseases except asthma such as pulmonary fibrosis, extensive bronchiectasis and cystic fibrosis, previous excision of one or more lobes by surgery, lung volume reduction surgery, active cancer under therapy, suspected lung cancer, chest radiation therapy, metallic objects in the chest and lung masses. Those with recent acute coronary event, recent chest or abdominal surgery, were pregnant, had multiple self-described race, or had first or second degree relative already enrolled in the study, were not enrolled. Subjects who had an exacerbation within the month prior to testing were also excluded. ***Controls:*** Normal non-smokers from a second study that prospectively obtained CT data (Image and Model Based Analysis of Lung Disease: NIH HL-064368) at the University of Iowa were included. This second study was an NIH funded bioengineering research partnership grant to develop new CT imaging techniques for pulmonary analysis, and involved high quality, spirometry controlled CT data acquisition of non-smoking subjects. Subjects were normal healthy volunteers between the ages of 20 and 90 years, who smoked not more than 20 cigarettes in their lifetime, and had normal spirometry. Those with body mass index more than 32 kg/m^2^, metal in the chest, pregnant or nursing females and those with asthma or a family history of COPD were excluded.

**Imaging:**

All CT data collected were obtained from a single Siemens Somatom Sensation 64 CT scanner (Siemens Healthcare, Erlangen, Germany) residing within a dedicated pulmonary imaging research facility. The COPDGene protocol consisted of gathering two fixed lung volume scans for quantitative lung analysis, at maximal full inspiration (total lung capacity, TLC) and normal end expiration (functional residual capacity, FRC). Subjects were coached by the CT technologist via voice commands to achieve the proper lung volumes. Lung volumes during scans obtained under HL-064368 were verified through use of a spirometer during scanning and FRC was defined as the lung volume at 20% vital capacity. The COPDGene scan protocol consisted of using 200 effective mAs for inspiratory and 50 mAs for expiratory scans, 120kV, pitch of 1.0, 0.75mm slice thickness and 0.5mm slice interval. The reconstruction kernels were B30 & B31 respectively. The scan protocol for the HL-064368 consisted of using 100 effective mAs, 120kV, 1.0 pitch, 0.75mm slice thickness, 0.5mm slice interval, B30, and B31 kernels. TLC, FRC, emphysema and gas trapping were analyzed using the Apollo software (VIDA Diagnostics, Coralville, IA). Lobar segmentation was achieved using an anatomy guided graph search method incorporated into the analysis software (Pulmonary Workstation, VIDA Diagnostics). As severe emphysema can challenge automated lobar segmentation, a rigorous quality control process combined with manual correction when required was employed to finalize lobar segmentation. The total segmented lung volumes, as well as the lobar boundaries, in the inspiratory and expiratory CT scans were used to calculate the TLC and FRC respectively for the whole lung and for lobar volumes. The percentage emphysema was calculated using the percentage of lung or lobe volume at TLC with attenuation less than -950 Hounsfield Units (HU). These were low attenuation areas (LAA950_insp_). The percentage gas trapping was calculated using the percentage of lung or lobe volumes at FRC with attenuation less than -856 HU (LAA856_exp_). Lobar predominance was defined by the lobe with the maximum percentage of LAA950_insp_ for emphysema and LAA856_exp_ for gas trapping. Segmentations of the trachea, main stem bronchi, segmental bronchi and two generations of sub-segmental bronchi were generated and subtracted from the segmented lung images for the calculation of LAAs.

**Pulmonary function testing (PFT):**

Spirometry was performed in cases (EasyOne spirometer, ndd, Zürich, Switzerland) and controls (OWL Body plethysmography, Ferraris Respiratory, Inc, Louisville, CO, or V6200 Body Box, Sensor Medics, verified for equivalency) according to the American Thoracic Society (ATS) guidelines. Spirometry was repeated 12-20 minutes following inhalation of two puffs of albuterol HFA with an appropriate spacer such as an Aerochamber® (Monaghan Medical Corporation, Plattsburgh, NY). Post bronchodilator values were used for diagnosis of COPD using a fixed cut-off of FEV_1_/FVC of <0.70.

**References**

1. Regan EA, Hokanson JE, Murphy JR, Make B, Lynch DA, et al. (2010) Genetic epidemiology of COPD (COPDGene) study design. COPD 7: 32-43.

2. Pauwels RA, Buist AS, Calverley PM, Jenkins CR, Hurd SS (2001) Global strategy for the diagnosis, management, and prevention of chronic obstructive pulmonary disease. NHLBI/WHO Global Initiative for Chronic Obstructive Lung Disease (GOLD) Workshop summary. American journal of respiratory and critical care medicine 163: 1256-1276.

3. Fuld MK, Grout RW, Guo J, Morgan JH, Hoffman EA (2012) Systems for lung volume standardization during static and dynamic MDCT-based quantitative assessment of pulmonary structure and function. Academic radiology 19: 930-940.

4. Hoffman EA, Simon BA, McLennan G (2006) State of the Art. A structural and functional assessment of the lung via multidetector-row computed tomography: phenotyping chronic obstructive pulmonary disease. Proceedings of the American Thoracic Society 3: 519-532.

5. Ukil S, Reinhardt JM (2009) Anatomy-guided lung lobe segmentation in X-ray CT images. IEEE transactions on medical imaging 28: 202-214.

6. Tschirren J, Hoffman EA, McLennan G, Sonka M (2005) Segmentation and quantitative analysis of intrathoracic airway trees from computed tomography images. Proceedings of the American Thoracic Society 2: 484-487, 503-484.

7. Miller MR, Hankinson J, Brusasco V, Burgos F, Casaburi R, et al. (2005) Standardisation of spirometry. Eur Respir J 26: 319-338.
